# Management of mucoid degeneration of the anterior cruciate ligament: a systematic review

**DOI:** 10.1186/s43019-021-00110-6

**Published:** 2021-08-21

**Authors:** Tamer Sweed, Mohamed Mussa, Ahmed El-Bakoury, Guido Geutjens, Andrew Metcalfe

**Affiliations:** 1 University Hospitals of Derby and Burton, Uttoxeter Rd, Derby, DE22 3NE UK; 2 Trauma and Orthopaedic Speciality Trainee, West Midlands Deanery, Birmingham Rotation, Birmingham, UK; 3 University Hospitals Plymouth NHS Trust, Plymouth, UK; 4 University of Alexandria, Alexandria, Egypt; 5 University Hospital Coventry and Warwickshire, Clifford Bridge Road, Walsgrave, Coventry, CV2 2DX UK

**Keywords:** Anterior cruciate ligament, Mucoid degeneration, ACL

## Abstract

**Purpose:**

The purpose of this study was to investigate the outcomes of management of mucoid degeneration of the anterior cruciate ligament (MDACL) by performing a systematic review of methods of treatment that have been reported.

**Methods:**

A systematic literature search in the databases MEDLINE, Embase, Google Scholar, Cochrane, ISI web of science and Scopus was performed through July 2020 by three independent reviewers. The review was performed according to the Preferred Reporting Items for Systematic Reviews and Meta-analyses (PRISMA) guidelines and registered in the PROSPERO database (CRD42018087782). Quality was assessed using the Methodological Index for Non-Randomized Studies (MINORS) criteria.

**Results:**

A total of nine studies were eligible for review. All nine studies assessed the outcome of arthroscopic debridement of MDACL. A total of 313 knees in 292 patients were included. The mean follow up ranged from 13 to 72 months. There was strong association between MDACL and chondral lesions (82%) and between MDACL and meniscal tears (69%). The rate of simultaneous meniscectomy ranged from 13 to 44%. Postoperative pain relief ranged from 53.8 to 95%. There was an improvement in postoperative range of motion and outcome scores (Lysholm and International Knee Documentation Committee scores and the Knee Injury and Osteoarthritis Outcome Score). Postoperative Lachman test was positive in 40% of patients, and 6% of patients had symptomatic instability. The mean MINORS score was 9.5 out of 16 (4–12).

**Conclusions:**

Arthroscopic debridement of the anterior cruciate ligament (ACL) results in satisfactory pain relief and improvement in knee outcome scores. Postoperative ACL laxity is common after arthroscopic ACL debridement, however, symptomatic instability is not. The need for delayed ACL reconstruction should be discussed preoperatively, especially if complete resection of the ACL is to be performed.

**Level of evidence:**

IV

## Introduction

Mucoid degeneration of the anterior cruciate ligament (MDACL) is a rare entity, first described by Kumar et al. in 1999 [1]. The aetiology of MDACL is not fully understood, hence, there are multiple theories on this. The origin of MDACL may be degenerative or traumatic. Another theory is the “synovial theory” whereby a pouch of synovium herniates and is subsequently filled with synovium [2].

MDACL is differentiated from synovial cysts of the anterior cruciate ligament (ACL) where, in MDACL, mucoid tissue intermingles within ACL fibres and is not contained within a cyst [3]. The prevalence of MDACL on magnetic resonance imaging (MRI) ranges from 1.8 to 5.3% [4, 5]. However, MDACL is asymptomatic in most patients. Patients with symptomatic MDACL commonly present with posterior knee pain and limitation of knee flexion or extension [2, 6].

MDACL is diagnosed by MRI, showing a celery stalk sign, and is confirmed by tissue biopsy and histological examination [7]. Bergin et al. have described the following MRI criteria [4]: (1) high signal intensity in the T1 and T2 sequences, (2) increased ACL volume and (3) continuous fibres of ACL shown in the T2 sequence. Arthroscopic diagnostic criteria [7] are (1) continuous ACL fibres, (2) increased ACL volume, (3) yellowish-coloured material expressed on palpation and (4) loss of ACL synovial lining. Histologically, there is a mucoid substance in connective tissue containing glycoproteins and mucoproteins [8].

Treatment for MDACL usually starts non-surgically with anti-inflammatory drugs and physiotherapy. Surgical treatment involves arthroscopic debridement of ACL, with partial or total resection, occasionally combined with ACL reconstruction. The success of treatment for MDACL and the subsequent risk of instability is not known. The purpose of this study was to investigate the outcome of management of MDACL by performing a systematic review of methods of treatment that have been reported. This study hypothesized that arthroscopic debridement of MDACL would provide patients with improvement in pain, range of movement and functional outcome scores.

## Materials and methods

We followed the Preferred Reporting Items for Systematic Reviews and Meta-Analyses (PRISMA) 2009 checklist [9]. This study was registered on PROSPERO [10], an international prospective register of systematic reviews (CRD42018087782).

### Search strategy

MEDLINE, Embase, Google Scholar, Cochrane, ISI web of science and Scopus were comprehensively searched from the earliest year of indexing until 10 July 2020. Three reviewers (an experienced librarian and two of the authors) independently searched these databases. Keywords used for the Search were “mucoid degeneration”, “anterior cruciate ligament” and “ACL”. Open Grey database was searched for grey literature using the same keywords.

### Eligibility criteria

The inclusion criteria for this study were (1) studies including symptomatic patients with mucoid degeneration of the ACL, (2) studies of 10 or more patients, (3) a minimum of 6 months follow up and (4) patients treated conservatively or surgically (arthroscopic debridement with or without simultaneous ACL reconstruction). Exclusion criteria were (1) case reports, literature reviews and conference abstracts and (2) studies written in languages other than English.

### Critical appraisal

Methodological quality of the studies was assessed using the Methodological Index for Non-Randomized Studies (MINORS) criteria [11] (Table 1). For non-comparative studies, this consists of eight questions. Each study was scored 0–2 on each question, with a global ideal score of 16. Items are scored as 0 (not reported), 1 (reported but inadequate) or 2 (reported and adequate). Categories were determined in accordance with the study of Ekhtiari et al. as “very low” (0–4 points), “low” (5–8 points), “good” (9–12 points) or “excellent” (13–16 points) [12].

**Table 1:** The revised and validated version of the Methodological Index for Non-Randomized Studies (MINORS)

Question	0	1	2
1. A clearly stated aim			
2. Inclusion of consecutive patients			
3. Prospective collection of data			
4. Endpoints appropriate to the aim of the study			
5. Unbiased assessment of the study endpoint			
6. Follow-up period appropriate to the aim of the study			
7. Loss to follow up less than 5%			
8. Prospective calculation of the study size			

The items are scored 0 (not reported),1 (reported but inadequate) or 2 (reported and adequate). The global ideal score is 16 for non-comparative studies

### Data extraction

Two reviewers independently screened the titles and abstracts of the retrieved records for eligibility. Any discrepancy was resolved by the senior author. Data extracted included mean age and gender of the patients, duration of follow up, associated chondral and meniscal lesions, interventions and outcomes.

### Data analysis

The extracted data were assessed for meta-analysis. The extracted data were heterogeneous in terms of outcome assessment. In the nine included studies, four different scores were used for outcome assessment. Due to the heterogeneity of data, a meta-analysis was not possible, and a narrative review was deemed most appropriate.

## Results

### Search results

**Fig. 1:**
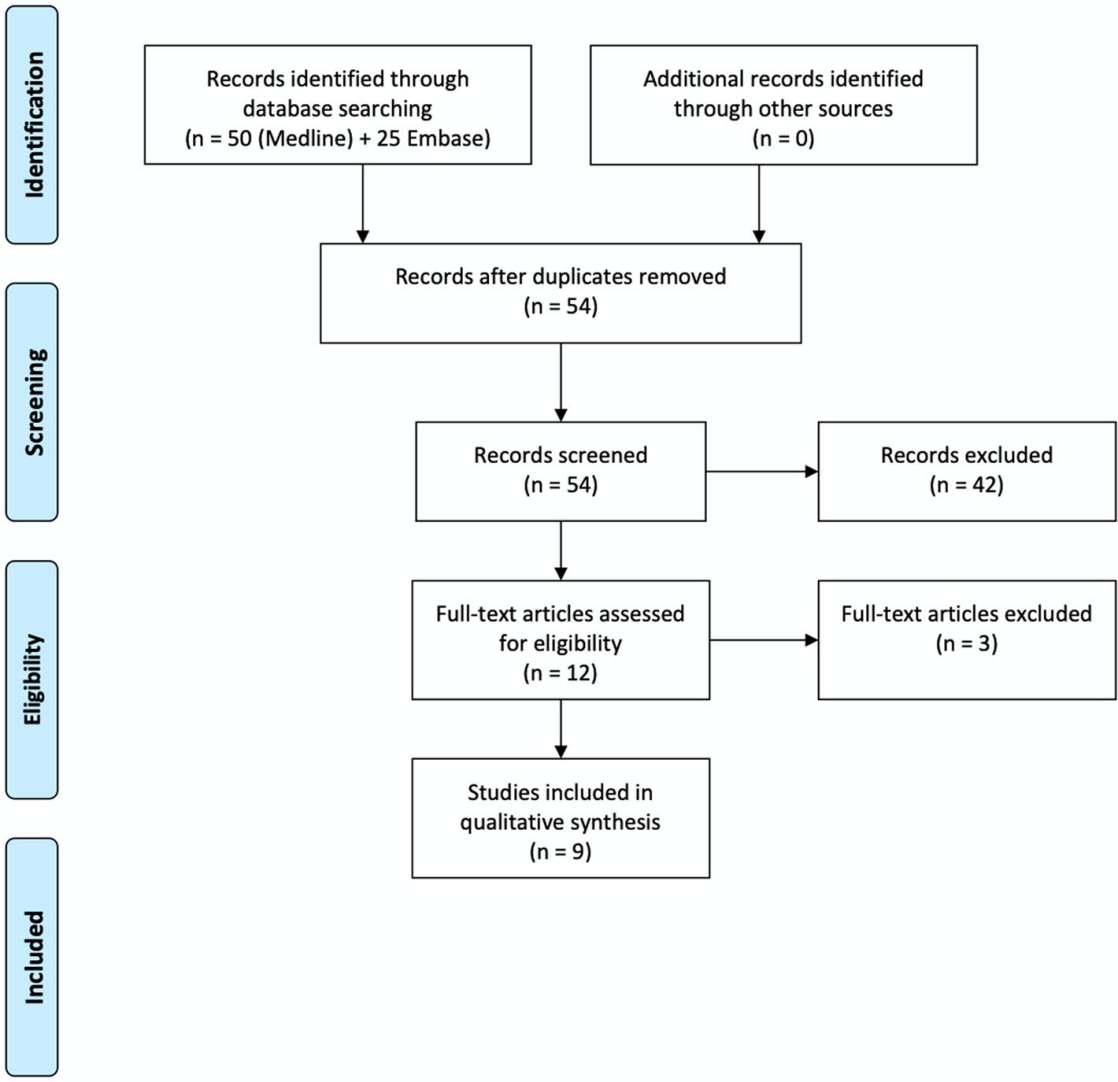
Preferred Reporting Items for Systematic Reviews and Meta-analyses (PRISMA) flowchart illustrating the selections of trials included in the systematic review

After the literature search and exclusion of duplicates, 54 papers were retrieved: 12 articles were assessed for eligibility, and of these, 9 papers met the inclusion criteria and were included in the systematic review [3, 6, 13–19] (Fig. 1).

### Assessment of methodological quality

All nine studies were case series; six were retrospective and three were prospective studies. None of the studies was a randomised controlled trial and none had a control group. The mean MINORS score was 9.5 (range 4–12). The quality of methodology was excellent in one study, good in five studies, low in two studies and very low in one study (Tables 1 and 2).

**Table 2:** Assessment of the methodological quality of the studies using Methodological Index for Non-Randomized Studies (MINORS)

Study	Q1	Q2	Q3	Q4	Q5	Q6	Q7	Q8	Total
Ventura, 2018 [19]	2	2	0	2	0	2	0	0	8
Srivastava, 2016 [15]	0	0	0	2	0	2	0	0	4
Pandey, 2014 [13]	2	2	2	2	0	2	2	0	12
Cha, 2013 [6]	2	2	2	2	0	2	1	0	11
Morice, 2013 [17]	2	0	0	2	0	2	0	0	6
Chudasama, 2012 [14]	2	0	2	2	0	2	2	0	10
Lintz, 2010 [3]	2	2	2	2	0	2	0	2	12
Kim, 2008 [16]	2	2	2	2	0	2	1	2	13
Khanna, 2016 [18]	2	2	2	2	0	2	0	0	10

### Study characteristics

A total of 313 knees in 292 patients were included in the nine studies. All nine studies included patients with symptomatic MDACL confirmed by MRI (Table 3). Patients in all studies presented with central or posterior knee pain, mainly with terminal extension in three studies [14, 16, 17]. The reported restriction of range of movement was flexion deficit in four studies [3, 13, 15, 17], extension deficit in one study [14] and combined flexion and extension deficit in four studies [6, 16, 18, 19] (Table 4).

**Table 3:** Characteristics of the studies included in the systematic review

Study	Study design	Knees	Mean age	% Female	Follow up (months)	Biopsy %	Chondral lesions %	Meniscal tears %	Intervention	Meniscectomies %
Ventura, 2018 [19]	Retrospective cohort	25	57 (37–64)	67	53 (37–64)	100	44	32	AD	32
Srivastava, 2016 [15]	Retrospective cohort	18	41 (27–60)	72	36 (12–59)	100	55	22	AD	NR
Pandey, 2014 [13]	Prospective cohort	11	40 (21–59)	45	13.8 (6–28)	100	63	9	AD	NR
Cha, 2013 [6]	Retrospective cohort	68	51 (35–75)	72	22 (12–20)	85	89	49	AD +/− notchplasty	44
Morice, 2013 [17]	Retrospective cohort	23	50 (31–70)	19	32 (8–70)	NR	39	61	Reduction plasty	13
Chudasama, 2012 [14]	Prospective cohort	20	42 (28–52)	40	24 (12–36)	100	55	30.5	AD +/− notchplasty	20
Lintz, 2010 [3]	Retrospective cohort	29	49 (28–68)	30	72 (12–120)	62	69	72	AD +/− notchplasty	38
Kim, 2008 [16]	Retrospective cohort	106	61 (42–80)	95	43 (25–74)	1	96	96	AD +/− notchplasty	NR
Khanna, 2016 [18]	Prospective cohort	13	36 (27–46)	62	8.4 (6–12)	100	NR	NR	AD +/− notchplasty	23

*AD* Arthroscopic debridement, *NR* Not reported

**Table 4:** Summary of clinical outcomes for studies included in the systematic review

Study	Outcome score	Preop average	Postop average	Preop ROM deficit (patient %)	Postop ROM deficit (patient %)	Positive Lachman %	Positive Pivot %	Symptomatic instability %
Ventura, 2018 [19]	Lysholm IKDC VAS	47 27 8 (6–10)	85 (65–99) 81 (56–87) 2 (0–3)	100% flexion deficit 16% extension deficit	23.3° average improvement	100%	0	NR
Srivastava, 2016 [15]	Lysholm	NR	87.2 (85–95)	100% flexion deficit	0%	16	0	0
Pandey, 2014 [13]	Lysholm	NR	89.5 (85–95)	63% flexion deficit	0%	72	0	0
Cha, 2013 [6]	Lysholm	50	83	53% flexion deficit 82% extension deficit	3% 7%	10	6	5.8
Morice, 2013 [17]	IKDC KOOS	NR NR	81 (45–97) 88 (56–99)	95% flexion deficit	13%	17	NR	0
Chudasama, 2012 [14]	IKDC	33.6	73.2	20% extension deficit	0%	70	10	0
Lintz, 2010 [3]	IKDC KOOS	NR NR	71 (42–92) 78 (26–99)	48% flexion deficit	21° average improvement	62	27.5	48.2 (2 delayed ACL reconstruction)
Kim, 2008 [16]	VAS	6.1	1.4	22% flexion deficit 78% flexion deficit	8% 14%	All firm end point	NR	0
Khanna, 2016 [18]	IKDC	36.3	73.1	92% flexion deficit 8% extension deficit	NR	NR	NR	0

*Preop* Preoperative, *Postop* Postoperative, *ROM* Range of motion, *NR* Not reported, *IKDC* International Knee Documentation Committee, *KOOS* Knee Injury and Osteoarthritis Outcome Score, *VAS* Visual analogue scale, *ACL* Anterior cruciate ligament

The mean age of patients ranged from 31 to 78 years. The retrospective study by Kim et al. had the largest number of patients (91) with a mean age of 61 years, which was the oldest study population amongst the nine studies [16]. The mean follow up ranged from 13 to 72 months. There was more than 20% loss to follow up in the study of Kim et al., while the three prospective studies had no loss to follow up [13, 14, 18]. Loss to follow up was not mentioned in the other five studies [3, 6, 15, 17, 19]. All nine included studies utilized MRI of the knee to establish a radiological diagnosis as an inclusion criterion. Histological diagnosis was established by tissue biopsy in all patients in five studies, with a total of 87 knees. Tissue biopsy was performed in 85% of patients (58 of 68) in the study of Cha et al. [6], in 62% of patients (18 of 29) in the study of Lintz et al. [3] and in 1% of patients (3 of 106 knees) in the study of Kim et al. [16]. Morice et al. [17] do not mention whether any patients underwent biopsy (Table 3).

### Associated lesions

There was a high rate of association between degenerative lesions and MDACL, ranging from 39 to 96%. The rate of association between meniscal tears and MDACL ranged from 9 to 96%. Khanna et al. [18] did not comment on an association between MDACL and chondral or meniscal lesions: after the exclusion of that report, the total number of patients with associated chondral lesions was 226 out of 275 patients (82%) and 190 patients had meniscal lesions (69%).

### Intervention

The primary intervention was arthroscopic ACL debridement in eight studies; this was combined with notchplasty in cases of impingement with the lateral wall of the notch in five studies [6, 14, 16–18] (Table 3). Morice et al. proposed a different intervention called reduction plasty, whereby the hypertrophic ACL is reduced to normal size without excision of all mucoid substance [17]. ACL debridement was partial in all studies except for those by Lintz et al. and Ventura et al. In the Lintz study, total resection of the ACL was performed in 17 out of 29 knees, while in the Ventura study total resection was performed in 17 out of 25 patients. Chudasama et al. report that one patient had ACL reconstruction simultaneously after the ACL debridement.

In six of the nine studies [6, 14, 16–18], arthroscopic debridement of the ACL was associated with simultaneous partial meniscectomy; this ranged from 13% in the study of Morice et al. to 44% in the study of Cha et al. (Table 3). There was no mention in the other three studies [13, 15, 18] of whether other procedures were performed simultaneously.

### Clinical outcomes

Clinical outcome was reported using a variety of scores (the Lysholm and International Knee Documentation Committee (IKDC) score, the Knee Injury and Osteoarthritis Outcome Score (KOOS) and the visual analogue scale (VAS) score) in the different studies; these are summarized in Table 4. Five studies demonstrated improved postoperative functional outcome scores compared to preoperative scores [6, 14, 16, 18, 19]. Four studies reported only postoperative scores, which were comparable to those in other studies. Eight studies showed postoperative improvement in range of motion, whereas Khanna et al. did not report on this. Postoperative pain relief was reported in seven studies. This ranged from 90 to 95% in all studies, except in the report by Kim et al., where only 53.8% of patients (57/106) had complete pain relief after surgery.

### Instability

Seven of the nine studies reported using the Lachman test to check postoperative ACL laxity. In these studies, the total number of patients with a postoperative positive Lachman test was 40% (77 of 194 patients). This was lowest in the study of Cha et al. (10%) and highest in the study of Ventura et al. (100%). However, symptomatic instability was not common. Eight studies reported postoperative instability. The total number of patients with symptomatic instability was 18 out of 288 (6%).

Symptomatic instability was highest in the study of Lintz et al. (48.2% (14/29 patients) with symptomatic instability), which may be due to the large number of patients with total resection of the ACL (58.6% (17 out of 29 patients)) (Table 4). Six studies reported using the pivot shift test; unsurprisingly, Lintz et al. reported the largest percentage of patients (27.5%) with a positive pivot test. Ventura et al. reported total resection of the ACL in 68% of patients: one young patient had disabling instability and subsequent ACL reconstruction. However, the authors did not report whether there were any other patients with symptomatic instability.

## Discussion

MDACL is not uncommon, however, it is rarely symptomatic [4, 5]. It is commonly identified on MRI in association with other pathological findings and can be confused with partial ACL tears. There is no consensus on treatment and evidence is scarce. To our knowledge, this is the first systematic review to look into the results of the management of MDACL.

In this systematic review we found that arthroscopic debridement resulted in good pain relief and improved range of motion and clinical outcome scores. There was strong association with chondral lesions and meniscal tears. This may support the theory that it is a degenerative process. Postoperative clinical laxity was common, however, symptomatic instability was not. All studies focused on the outcome of arthroscopic debridement of MDACL, with no studies on conservative treatment. Only three studies mentioned failed conservative treatment as an inclusion criterion [14, 15, 18].

The aetiology of MDACL is not clearly understood. In this review, the mean age of patients ranged from 31 to 78 years; mean age was highest in the study of Kim et al. (61 years), which was the largest study and represented more than one third of the total patients included in this review. There was strong association between degenerative changes and MDACL (82% having chondral and 69% meniscal lesions), which might suggest that MDACL is a degenerative process.

Arthroscopic debridement of the ACL was the intervention in eight studies, whereas Morice et al. proposed reduction plasty, which is reduction of the ACL to normal volume without excision of all mucoid tissue. This did not appear to affect the postoperative rate of clinically detectable laxity, which was comparable to that in other studies.

Arthroscopic debridement of the ACL resulted in good pain relief and improved range of movement in all studies, with postoperative improvement in the knee scores. However, in most studies a proportion of patients simultaneously underwent other arthroscopic procedures for associated pathological knee conditions (44% meniscectomies in the study of Cha et al.). This makes it difficult to relate the improvement of symptoms to ACL debridement. Furthermore, this raises the question as to whether the symptoms (pain and diminished range of movement) were solely due to MDACL and not secondary to other associated pathological conditions. As there was no control group in any of the studies and the natural history of MDACL is not known, the postoperative improvement may have been attributed to the placebo effect, simultaneous arthroscopic procedures or the postoperative rehabilitation, or may be due to the pain having resolved without treatment at all.

Despite the low rate of complications in all studies, there was a high incidence of postoperative knee laxity, demonstrated by a positive Lachman test, in most studies. Postoperative symptomatic instability was not common in eight studies, but was observed in 48% of patients in the study of Lintz et al., with two patients having a delayed ACL reconstruction. The need for simultaneous or delayed ACL reconstruction should be discussed with patients in case total resection of the ACL is to be performed, particularly in young active patients.

This systematic review has some limitations. First, our literature search revealed a heterogeneous group of studies dominated by case reports and case series, with no randomised controlled trials. Only nine studies met our inclusion criteria and they were all case series. Second, methodological quality assessment showed that three studies had low methodological quality. Third, clinical outcomes were reported using a variety of scores, so a data meta-analysis was not possible. Finally, partial meniscectomy was commonly performed simultaneously with ACL debridement in all studies, which arguably could be the reason for the postoperative improvement.

## Conclusion

Arthroscopic debridement of the ACL results in satisfactory pain relief and improvement in knee outcome scores. Postoperative ACL laxity is a common finding after ACL arthroscopic debridement, however, symptomatic instability is not. The need for delayed ACL reconstruction should be discussed preoperatively, especially if complete resection of the ACL is to be performed. There is strong association between MDACL and chondral and meniscal lesions, which may support the theory that MDACL is a degenerative process.

## Data Availability

Available on request.
